# Inhibition of Zoonotic Pathogens Naturally Found in Pig Manure by Black Soldier Fly Larvae and Their Intestine Bacteria

**DOI:** 10.3390/insects13010066

**Published:** 2022-01-07

**Authors:** Osama Elhag, Yuanpu Zhang, Xiaopeng Xiao, Minmin Cai, Longyu Zheng, Heather R. Jordan, Jeffery K. Tomberlin, Feng Huang, Ziniu Yu, Jibin Zhang

**Affiliations:** 1 State Key Laboratory of Agricultural Microbiology, National Engineering Research Center of Microbial Pesticides, College of Life Science and Technology, Huazhong Agricultural University, Wuhan 430070, China; osamamama6@hotmail.com (O.E.); yuanpu201@126.com (Y.Z.); xiaopeng307@126.com (X.X.); cmm114@mail.hzau.edu.cn (M.C.); ly.zheng@mail.hzau.edu.cn (L.Z.); fenghuang@mail.hzau.edu.cn (F.H.); yz41@mail.hzau.edu.cn (Z.Y.); 2 Faculty of Science and Technology, Omdurman Islamic University, Khartoum 11111, Sudan; 3 Department of Biology, Mississippi State University, Starkville, MS 39762, USA; jordan@biology.msstate.edu; 4 Department of Entomology, Texas A&M University, College Station, TX 77843, USA; jktomberlin@tamu.edu

**Keywords:** *Hermetia illucens* L., antimicrobial peptide, gut-isolated bacterium, *Staphylococcus aureus*, *Salmonella*

## Abstract

**Simple Summary:**

With the rapid development of the economy and the improvement of people’s living standards, people need to rear a lot of livestock to meet demand for proteins. This also involves an increase in the production of livestock manure. The expanding rate of livestock manure has become a thorny issue, owing to characteristics such as plentiful nitrogen and abundant zoonotic pathogens. The saprophagous larvae of the black soldier fly (BSF) are often associated with animal manure and can significantly reduce the populations of different zoonotic pathogens in livestock manure. However, reports about the mechanisms of this phenomenon are scarce. In this study, we investigated the potential mechanisms of BSF larvae in reducing the zoonotic pathogens naturally found in pig manure. The results clearly showed that zoonotic pathogens in pig manure were significantly decreased after being treated with BSF larvae, and also suggested that the antimicrobial peptides produced by the BSF larvae and gut-associated bacteria are able to antagonize the zoonotic pathogens. This study will contribute to reveal the potential antagonistic mechanisms of BSF larvae against zoonotic pathogens and improve the safety of organic waste conversion by BSF larvae.

**Abstract:**

Black soldier fly (BSF) larvae are often exposed to organic waste which harbors abundant zoonotic pathogens. We investigated the ability of BSF larvae to inhibit the zoonotic pathogens naturally found in pig manure. The zoonotic pathogens populations were detected by using selective medium during the conversion. Results showed that the viability of the zoonotic pathogens in pig manure was significantly affected. After eight days of conversion, the *Coliform* populations were undetected, and *Staphylococcus aureus* and *Salmonella* spp. decreased significantly on the eighth day. Antimicrobial assays of the purified recombinant defensin-like peptide 4 (DLP4) showed that this peptide exhibits inhibitory activity against *S. aureus*, *Salmonella enterica* serovar typhimurium, and *Escherichia coli* in vitro. Bacteria BSF-CL and BSF-F were isolated from the larvae gut, and both inhibited the growth of *S. aureus* and *E. coli*, but *Salmonella* spp. was sensitive to the BSF-CL strain (but not to the BSF-F strain). The results from our experiments indicate that BSF larvae are capable of functionally inhibiting potential zoonotic pathogens in pig manure through a variety of mechanisms including antimicrobial peptides expression and the gut associate microorganisms. This study provides a theoretical basis for further study on the combined mechanism of BSF larvae immunity and its gut microbes against the zoonotic pathogens in pig manure.

## 1. Introduction

Insects are the largest group in the animal kingdom in terms of the number and diversity of species [1]. These species are often associated with animal manure, which harbors many significant food-borne pathogens [2]. The insect antimicrobial peptides (AMPs) are key factors of the immune system of insects, and they are mainly synthesized by the fat body, a large biosynthetic organ that is functionally similar to the mammalian liver [3]. Different antimicrobial substances are produced on the surface of insects to prevent microbial infection. The functional significance of the AMPs of insects in reducing the common pathogens in the surrounding environment has been demonstrated [4].

Insect diversity is reflected in the large and varied microbial communities inhabiting the gut [5]. These microorganisms contribute to the biological activity of their hosts and play a major role for stimulating the immune system and resistance against the invading pathogens [1]. In addition, some insect species provide ideal conditions for bacterial conjugation, which suggests that the gut is a “hot spot” for gene transfer [6]. Genomic analysis provides new avenues for the study of the gut microbial community and reveals the molecular foundations of the relationships between the insect and its microbiome [7]. Indigenous gut bacteria also play a role in withstanding the colonization of the gut by nonindigenous species, such as pathogens [8]. Gut bacteria have been documented to show antagonistic activity against pathogenic bacteria and fungi [9].

The defensin and defensin-like AMP family have been identified in insect, plant, and mammalian. These protein species play an important role in the innate immune systems of those organisms and mainly affect Gram-positive bacteria and appear to be the abundant group throughout the inducible AMP [10]. Recently, a novel insect defensin-like peptide DLP4 is isolated and identified in immunized hemolymph and various tissues of BSF; it shares high sequence similarity (60–67.5%) to other insect defensins, such as sapecin, and lucifensin [11,12,13]. Structurally, defensin and defensin-like AMPs have a common conserved three intermolecular disulfide bridges motif with a molecular size of 3–4 kDa [14]. Ubiquitin was first isolated from bovine thymus as an adenylate cyclase stimulating polypeptide [15].Ubiquitin is a highly conserved polypeptide consisting of 74–76 amino acid residues with a molecular weight of 8.4–8.5 kDa. This molecule is found in all groups of organisms from protozoan to vertebrates [16]. In eukaryotic organisms, ubiquitin is known as the protein degradation marker through the ubiquitin-proteasome pathway [17]. Invertebrates have additional functions, such as cellular relocating, stability or activity of proteins, and as recently found, antimicrobial activities [18].

In nature, animal manure is the main food for many insects, which are able to efficiently convert manure into biomass and one of the most environmentally friendly and most economically viable insect species is the black soldier fly (BSF, *Hermetia illucens*; Diptera: Stratiomyidae) [19]. BSF larvae can efficiently convert the kitchen waste, spoiled feed, manure, and garbage. Thus, this species is in close contact with pathogenic microorganisms from larvae to adult [20], but has a defense mechanism to infection [21]. The BSF larvae have been reported to significantly reduce the populations of different zoonotic pathogens within the livestock and dairy manures [22,23]. Furthermore, various antimicrobial properties and substances of the BSF larvae have been reported [11,24,25]. The strong antimicrobial activity of the immunized hemolymph of the BSF larvae was confirmed against three different *Salmonella* species [26]. Zhou et al. have isolated bacterial strains from the gut of BSF larvae and confirmed that gut-associated *Bacillus subtilis* BSF-CL exhibits strong inhibitory effect against fungus *Xanthomonas oryzae* PXo99 and *Rhizoctonia solani* AG-8 [27].

We hypothesized that AMPs and the gut-associated microorganisms of the BSF larvae play essential roles in reducing the zoonotic pathogen populations naturally found in manure. To test this hypothesis, we fed pig manure to the BSF larvae and evaluated the BSF larva-associated antimicrobial activity against naturally present zoonotic pathogenic bacteria in the manure. We explored the underlying potential mechanisms by examining the expression patterns of defensin-like peptide 4 (DLP4) and Cg-ubiquitin AMP genes in the BSF larvae. The DLP4 was subsequently selected for large-scale production to investigate the antimicrobial spectrum of the molecule against some zoonotic pathogenic bacterial strains isolated from pig manure. Finally, we isolated microbes from the guts of the BSF larvae to identify their function against zoonotic pathogens in vitro. This work aimed to establish the relationship between the intestinal bacteria and antimicrobial peptides of BSF larvae and zoonotic pathogens naturally in pig manure.

## 2. Materials and Methods

### 2.1. Rearing of Neonate BSF Larvae

Neonate BSF larvae of Wuhan strain were obtained from a colony maintained year-round in a greenhouse [28] at the State Key Laboratory of Agricultural Microbiology, Huazhong Agricultural University in Wuhan, China. The colony was maintained in a greenhouse at 27 ± 1 °C with 60–70% relative humidity and 12 h light/12 h dark photoperiod.

### 2.2. Pig Manure

Fresh pig manure was collected from the National Engineering and Technology Research Center for Livestock of Huazhong Agricultural University, Wuhan, China before being flushed using water.

### 2.3. BSF Larva Conversion Systems

BSF larvae were fed with a diet (50% wheat bran and 50% corn flour) with 65% water content at 28 °C and 65% humidity for the first six days. Feeding was stopped on the seventh day, and larvae were separated from the feed. Then a total of 7500 seven-day-old BSF larvae with approximately the same weight were added to 5 kg fresh pig manure with 70% water content in a plastic container (length: 25 cm, width: 20 cm, height: 12 cm) and labelled as ‘pig manure inoculated larva group’ (PM + BSFL). The control pig manure did not contain larvae and labelled as ‘pig manure group’ (PM). Each treatment was set up in three replicates and kept for eight days at 28 °C and humidity of 65% in the green house.

### 2.4. Zoonotic Pathogen Populations Counting Methods

The dynamic changes of zoonotic pathogens were detected during the transformation of pig manure by BSF larvae. The zoonotic pathogen populations were counted as previously described [29]. In brief, manure samples (10 g) at different time points (zero, two, four, six, and eight) were collected from each container by using multipoint sampling method, each point collected 2 g manure. Then manure samples mixed with 90 mL of sterile physiological saline in a sterile 250 mL conical flask and shaken for 15 min at 180 rpm. The sample was made to stand for 5 min at room temperature, and the upper layer was withdrawn for the subsequent tests. The supernatant was pipetted for 10-fold gradient dilution. Crystal violet neutral red bile salt agar medium (VRBA), Baird-Parker medium, Bismuth sulfite (BS) agar medium were purchased from Qingdao Hope Bio-technology Co., Ltd. (Qingdao, China) and used as a selective and differential culture medium for *Coliforms*, *S. aureus* and *Salmonella* spp. counting, respectively. About 100 μL of each gradient dilution was pipetted and coated with selective medium. Each gradient dilution was set up in three replicates on each selective medium and cultured in a 37 °C constant temperature incubator for more than 36 h. The bacterial numbers of the three zoonotic pathogens were recorded. The number of the colony on the plate around 30–300 CFU was used to take count of the total bacteria.

### 2.5. Screening of the Antagonistic Activity of the Bacteria from the Gut of the BSF Larvae against Manure-Associated Zoonotic Pathogens

The intestinal bacterial were isolated from the gut of BSF larvae after eight days conversion, the larvae were separated from the fecal and disinfected with 75% alcohol for 3 min and rinsed with sterile water three times. The intestinal extract was prepared after dissection and homogenization. Then, the intestinal extract was diluted by using sterile water. Luria-Bertani (LB) agar (tryptone [10.0 g/L], yeast extract [5.0 g/L], NaCl [10.0 g/L], agar [20.0 g/L], and 1 L of distilled water, pH 7.0 ± 0.2) was used to isolate and purify the bacterial strains from each gradient dilution. The antagonistic effect of the BSF gut isolates were tested on Petri dishes by the dual culture method [30] against three original zoonotic pathogenic microorganisms from the pig manure. All isolates were cultured for two days on the LB plates. *S. aureus*, *E. coli*, and *Salmonella* spp. were grown to logarithmic phase at 37 °C in the LB broth. Then, 10 µL aliquot of each of the cultures was diluted in 10 mL of preheated LB containing 0.7% agar. The bacterial plugs (5 mm diameter) of the gut isolate antagonists were placed at the center of each plate surface, and then the plates were incubated at 30 °C for 24 h. PCR amplication of the functional bacterial 16S rRNA genes was performed using the forward primer 27F (5′-GTTTGATCCTGGCTCAG-3′) and the reverse primer 806R (5′-GGTTACCTTGTTACGACTT-3′). The PCR products were sent to Tsingke Biotechnology Co., Ltd. (Wuhan, China) for sequencing.

### 2.6. Quantitative Real-Time PCR (qRT-PCR) Analysis of the AMP Genes

Three larvae were collected on days zero, two, four, six, and eight from each container and soaked in 75% alcohol for 30 min. Then, the larvae were washed with RNase-free sterile water thrice and treated with liquid nitrogen. The total RNA was extracted using Trizol and determined on 1% agarose gel electrophoresis. Then, 5 × HiScript^®^ III qRT SuperMix (Nanjing Vazyme Biotech Co., Ltd., Nanjing, China) was used to synthesize the cDNA. Moreover, conventional PCR was run for β- actin, DLP4, and Cg-ubiquitin. The amplified gene was analyzed by utilizing 2% electrophoresis gel and sequenced. After the PCR results and DNA sequence analysis confirmed that the amplified fragments represented the anticipated cDNA fragment of the tested and control genes, expression levels of the AMP transcripts were detected using the ABI 7500 (Applied Biosystems, Foster, CA, USA). The specific primers used are listed in Table 1. The relative expression of DLP4 and Cg-ubiquitin to the reference gene *β-actin* was calculated using the 2^−ΔΔCT^ method [31]. The data represented the mean of three independent reactions performed for each sample.

### 2.7. Strains, Plasmids, and Reagents

*Escherichia coli* DH5α, *Escherichia coli* CICC10003 *S. aureus* CICC10001, and *Salmonella enterica serovar* Typhimurium CICC10420 were obtained from the State Key Laboratory of the Agricultural Microbiology of Huazhong Agricultural University in Wuhan, China. *Pichia pastoris* GS115 and the yeast expression vector pGAPZαA were obtained from Invitrogen (Carlsbad, CA, USA). The codon-optimized DLP4 gene (120 bp) in the pGH vector was performed using the forward primer (5′-CCGCTCGAGAAAAGAGCTACCTGTGATTTGTTGTC-3′) and the reverse primer (5′-CGCTCTAGACCCTTTCTGCAGTTGCAGACAGC-3′) and provided by Tsingke Biotechnology Co., Ltd. (Wuhan, China). TA cloning vector pMD18-T, T4 DNA ligase, dNTPs, DNA Taq polymerase, restriction endonuclease enzymes, and DNA Marker were purchased from TaKaRa Biotechnology (Shiga, Japan). AxyPrep plasmid Miniprep Kit and AxyPrep DNA Gel Extraction Kit is the product of Axygen Scientific Inc (San Francisco, CA, USA). Trizol Reagent, RNase free ddH2O, 4× gDNA wiper, Mix 5× HiScript^®^ III qRT SuperMix, 2 × SYBR Green qPCR master mix Kit were the products of Vazyme Biological Technology (Wuhan, China). Diethylpyrocarbonate was obtained from Sigma-Aldrich (St. Louis, MO, USA). Oligonucleotide primers were designed using Primer Premier 5 and synthesized by Tsingke Biotechnology Co., Ltd. (Wuhan, China).

### 2.8. Assay of the DLP4 Antimicrobial Activity In Vitro

The pGAPZαA-DLP4 expression plasmid was constructed (Figure S2) and transformed into *Pichia pastoris* GS115 to express DLP4 in the YPD medium as previously described [32]. Then, the supernatant of the culture was harvested by centrifugation at 5000× *g* for 10 min. Precipitation was carried out with 80% ammonium sulfate. After desalting, Ni-NTA HisTrap crude column chromatography was used to purify the protein. The recombinant DLP4 was freeze-dried and assessed by Western blot with mouse anti-His (C-term) antibody after the protein was separated by Tricine SDS-PAGE [33].

*S. aureus* CICC10001, *S. enterica serovar* Typhimurium CICC10420, and *E. coli* CICC10003 were grown to stationary phase at 37 °C in Mueller Hinton (MH) broth. Cultures were then diluted with fresh MH broth to an OD_600_ of 0.001. To assay the antimicrobial activity, an equivalent amount of 80 mL of bacteria was distributed into the microplate containing 20 mL of water or the fraction to be analyzed. Bacterial growth was evaluated after 24 h at 30 °C by optical microscopy and after 48 h by measuring the culture absorbance at 600 nm. All experiments were performed in triplicate.

### 2.9. Data Analysis

Zoonotic pathogen populations were converted to log_10_ CFU/g before statistical analysis. All mean values with standard deviation were calculated for each bioindicator using Microsoft Excel. One-way ANOVA was used to determine the significant difference between groups, with the coefficient of significance set to *p* < 0.05, and Tukey’s multiple range tests were employed to perform multiple comparisons between means by using SPSS 22.0. Statistical significance was considered at *p* < 0.05.

## 3. Results

### 3.1. Evaluation of the Efficiency of the BSF Larvae in Reducing Zoonotic Pathogens in Pig Manure

**Figure 1:**
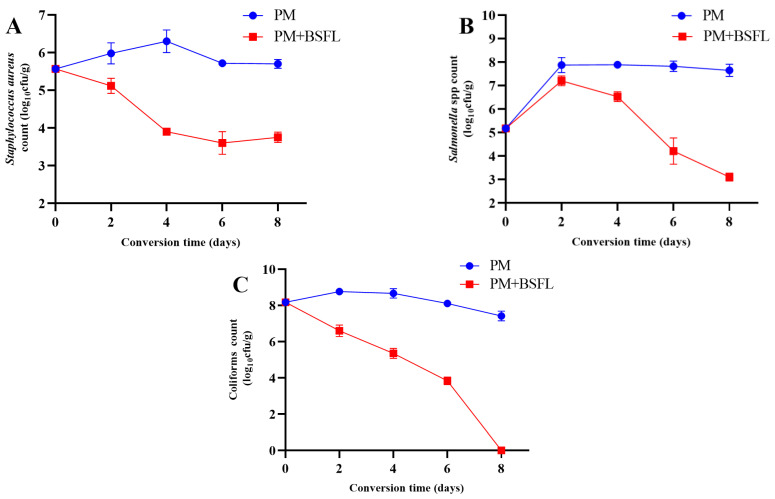
Black soldier fly (BSF) larval inactivation of *Staphylococcus aureus* (**A**), *Salmonella* (**B**), and *coliforms* (**C**) in pig manure. Each point represents the mean of three independent trials (n = 3).

Figure 1 illustrates the effectiveness of the BSF larvae in inhibiting the proliferation of the zoonotic pathogens. The populations of *S. aureus* in the larva-treated manure decreased from 5.57 log CFU/g on day 0 to 3.75 log CFU/g on day eight. Significant reductions were observed on days four, six, and eight (Figure 1A). *Salmonella* spp. populations in larva-treated manure increased on day two (7.20 log CFU/g) and day four (6.53 log CFU/g) and then decreased from day six (4.21 log CFU/g) to day eight (3.10 log CFU/g) (Figure 1B). The number of *coliforms* gradually decreased from 8.18 log CFU/g on day zero to 3.84 log CFU/g on day eight, but no bacteria was detected on day 8 (Figure 1C).

### 3.2. Antagonistic Activity In Vitro

**Figure 2:**
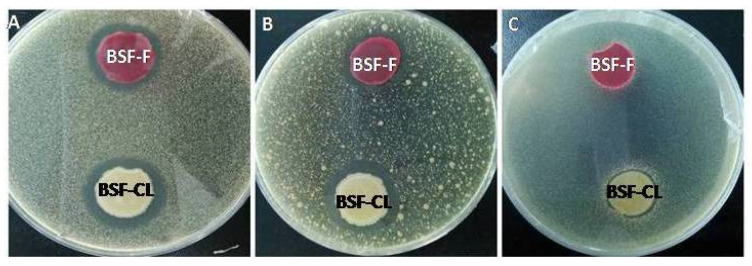
Bacteria from the guts of the BSF larvae inhibited the zoonotic pathogens *in vitro.* Strains BSF-CL and BSF-F inhibited (**A**) *S. aureus* and (**B**) *E. coli*; Strain BSF-CL inhibited (**C**) *Salmonella* spp.

A total of 11 strains have been isolated from the gut of the BSF larvae. Two strains coded BSF-F and BSL-CL showed clear antagonistic activity and identified as *Serratia marcescens* and *B. subtilis* (Table 2). The results showed that both antagonistic microorganisms inhibited the growth of *S. aureus* and *E. coli* with varying efficiencies (Figure 2A,B). *Salmonella* spp. was sensitive to the BSF-CL strain but not to the BSF-F strain (Figure 2C).

### 3.3. Transcriptional Patterns of Antibacterial Genes DLP4 and Cg-Ubiquitin

**Figure 3:**
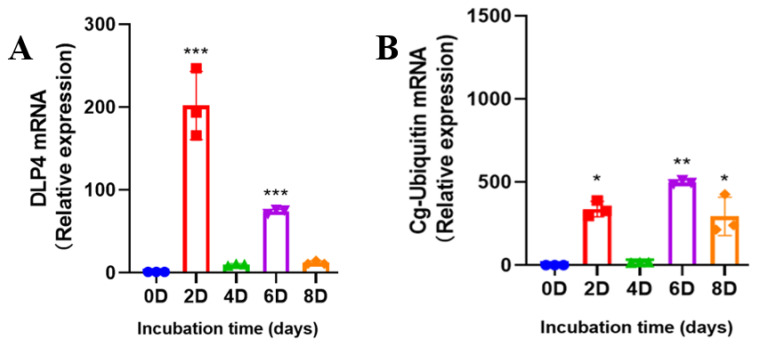
Quantitative real-time PCR analysis of DLP 4 and Cg-ubiquitin mRNA expression. (**A**) Temporal expression of the DLP4 transcripts in the larvae of BSF collected from the PM at different time points (zero, two, four, six, and eight days). (**B**) Temporal expression of Cg-Ubiquitin transcripts in the BSFL collected from PM at different time points (zero, two, four, six, and eight days). Statistical analyses were performed using ANOVA (* *p* < 0.05, ** *p* < 0.01, *** *p <* 0.001), and the data were analyzed compared with those on day 0.

To determine the mRNA expression profile of DLP4 and Cg-ubiquitin in the BSF larvae, total RNA samples from the larvae incubated in PM were analyzed by qRT-PCR, and the 2^−ΔΔCT^ method was applied to calculate the transcript levels of the AMP genes. The highest expression of DLP4 was found at 48 h (relative expression 202-fold) after inoculation in PM, but the transcription levels decreased on day four (relative expression 9-fold). Levels increased again on day six (relative expression 74-fold) and then decreased on day eight (relative expression 12-fold) (Figure 3A). The expression of Cg-Ubiquitin and DLP4 has the same trend but differ in their quantity production. The expression of Cg-Ubiquitin increased on day two (relative expression 337-fold) and then decreased on day four (relative expression 17-fold). Levels increased on day 6 (relative expression 498-fold) and decreased on day eight (relative expression 293-fold) (Figure 3B). However, the results showed that the expression of Cg-Ubiquitin was significantly greater and more sensitive than that of DLP4. The expression of DLP4 showed a stronger response in the early stages than during the later period, but Cg-Ubiquitin showed a greater response in the late stages.

### 3.4. Recombinant Expression and Purification of the DLP4 Gene in P. pastoris

**Figure 4:**
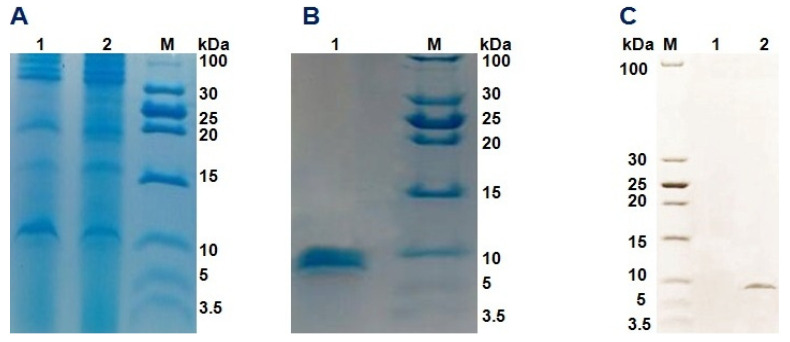
Tricine-SDS-PAGE and Western blot analysis of DLP4 expressed in *Pichia pastoris*. (**A**) Analysis of proteins of the broth supernatant from the crude culture of DLP4-expressing GS115; Lanes 1 and 2: the culture supernatant samples collected at 96 h and 120 h of incubation, respectively; M, low range unstained protein marker. (**B**) Analysis of the purified DLP4; Lane 1: Nickel affinity chromatograph of the purified-DLP4; M, low range unstained protein marker. (**C**) Western blot result of the purified-DLP4; M: low range unstained protein marker; lane 1: supernatant of GS115/pGAPZαA; lane 2: purified DLP4.

The *P. pastoris* colony with the highest DLP4 production was chosen for the large-scale peptide expression under optimal conditions, and the maximum secretion of recombinant DLP4 was observed after 96 h of incubation at 28 °C (Figure 4A). The His-tag in the N-terminus was affinity-purified using Ni–NTA materials. Approximately 15.8 mg of pure recombinant DLP4 was obtained from 500 mL of YPD culture. A 6.9 kDa single band was observed on the Coomassie blue-stained Tricine-SDS-PAGE gel as shown in Figure 4B. The expression efficiency was detected with immunoblot analysis, in which 100 μg/mL from the purified protein were applied into the Tricine-SDS-PAGE (16.5%) prior to the Western blot assay. The result revealed that the purified recombinant DLP4 was specifically recognized by the mouse anti-His (C-term) antibody as illustrated in Figure 4C.

### 3.5. Antimicrobial Assays of the Purified DLP4

Minimal inhibitory concentration (MIC) assays were carried out against the PM-associated Gram-positive bacteria and Gram-negative bacteria to evaluate the antimicrobial activity of the purified DLP4 expressed by *P. pastoris*. Recombinant DLP4 exerted strong inhibitory effects against *S. aureus* CICC10001 and *S. enterica* serovar Typhimurium CICC10420 but was less active against *E. coli* CICC10003 (Table 3).

## 4. Discussion

In this study, we examined the capability of BSF larvae to inhibit potential zoonotic pathogens in pig manure as well as to understand the influence of the modulation of AMP gene expression and antibacterial activity in the larvae. Different potential zoonotic pathogen strains, namely, *S. aureus*, *Salmonella* spp., and *Coliform* were analyzed in the pig manure after the BSF larvae were added. We determined that BSF larvae can reduce natural potential zoonotic pathogen populations in pig manure and the immune system of BSF larvae can produce antimicrobial peptides to inhibit potential zoonotic pathogens in pig manure. We also isolated two intestinal microorganisms named BSF-CL and BSF-F that could inhibit *S. aureus* and *E. coli* in vitro and BSF-CL have the function of inhibiting *Salmonella* in vitro.

As an ecological decomposer, the BSF larvae are often present in areas where they are in close contact with zoonotic pathogenic microorganisms, such as bacteria and fungi, from the larva to adult stages [34,35]. BSF larvae can survive well in these environments owing to competing with bacteria for nutrients or inhibiting them [13]. Moreover, the disinfection action of the BSF larvae may be achieved by ingesting and digesting microorganisms, as well as through the release of different types of antimicrobial peptides. BSF larvae have been successfully applied to convert different kinds of organic waste and had the ability to reduce zoonotic pathogen bacteria when treating organic wastes, especially *Salmonella* spp. [36]. In this study, the capability of the BSF larvae in eliminating the natural zoonotic pathogens in the pig manure was examined. Compared with control group, the populations of *S. aureus* and *Salmonella* spp. were significantly reduced in the larva-treated pig manure. The *Coliform* were undetected in pig manure after eight days conversion by BSF larvae. Intestinal microorganisms of BSF larvae may play a pivotal role in this progress. In recent years, a few of researchers focused on the composition of the BSF larvae gut microbiota through the culture-independent techniques (such as metataxonomics based on 16S rRNA gene sequencing) and analyzed the relationship between the BSF larvae gut microbiota and potential zoonotic pathogens [37,38]. However, in order to obtain pure cultures for research purposes and industrial applications, there is still a need to rely on culture methods, including incubation, isolation and identification steps [39]. In this study, we isolated two functional microorganisms from the gut of BSF larvae through incubation and isolation. They have the ability to antagonize zoonotic pathogens in vitro. These functional microorganisms may produce some antimicrobial active component in the BSF larvae gut and inhibit the growth of zoonotic pathogens.

Insects digesting manure are able to suppress pathogens by producing AMPs with a broad spectrum of activity against pathogen bacteria and/or fungi [40]. Most known AMPs from insects are antibacterial agents [41]. BSF larvae have sole properties that can be employed for various defense purposes and contain a range of AMPs as potent inhibitory substances against different zoonotic pathogens [2]. Furthermore, previous studies have indicated a close correlation between the resistance of the BSF larvae to zoonotic pathogens and the antimicrobial substances produced by the larvae [11,24,34]. However, additional studies are needed to explore the antimicrobial substance derived from the BSF larvae.

A comparative bioinformatics analysis of immunity-related transcriptomes from insects has revealed unexpected evolutionary plasticity [42]. The defensins are the dominant innate immune factors in different insect species and are generally overexpressed in many insects infected with microorganisms [10]. In this context, previous studies have been conducted to evaluate the antimicrobial activity of insect defensin and DLPs [11,43,44]. Among these molecules, DLP4 and Cg-ubiquitin were selected to identify their expression patterns in the BSF larvae. The analysis results of qRT-PCR revealed that the high expression of DLP4 and Cg-ubiquitin have positive correlation with the inhibition of *Coliform*, *S. aureus*, and *Salmonella* spp. In general, defensin demonstrates a strong antimicrobial property against different Gram-positive and Gram-negative bacteria [45]. The antimicrobial peptide DLP4 isolated from the BSF have advantages over the conventional antibiotics in reducing the multi-drug resistant methecillin-resistant *S. aureus* [11]. Studies on the bactericidal effect of blowfly-derived antimicrobial substances showed potential inhibitory activity against *S. aureus* [46,47].

Prior studies on antimicrobial peptides in BSF mostly focused on their expression and antibacterial activity in vitro, but little did pay attention to their expression in vivo. In this study, we investigated the antimicrobial peptides expression levels after inoculated into pig manure. We found the highest expression of DLP4 and Cg-Ubiquitin were found at 48 h and 144h, respectively. The expression of DLP4 showed a stronger response in the early stages than during the later period and Cg-Ubiquitin showed a greater response in the late stages, but the expression of Cg-Ubiquitin and DLP4 has the same trend. This may the BSF larvae needs to express DLP4 and Cg-Ubiquitin in response to current environmental stress, or these two genes are co-expressed in the larvae. Due to antimicrobial peptides are not only expressed when immune responses are triggered by invading pathogens. For example, the expression of particular AMP subsets in the burying beetle *Nicrophorus vespilloides* is gender-specific and regulated by the presence of offspring or carcass [48]. Thus, during the conversion by BSF larvae, it may the BSF larvae expressed antimicrobial peptides and inhibited zoonotic pathogens in pig manure.

It has been reported that the antimicrobial peptide DLP4 isolated from the BSF has advantages over the conventional antibiotics for reducing the multi-drug resistant methicillin-resistant *S. aureus* (MRSA) [11]. Studies investigating the bactericidal effect of blowfly-derived antimicrobial substances showed potential inhibitory activity against *S. aureus* [46,47]. For a better explanation of the antimicrobial properties of AMPs against manure-associated pathogens or other phytopathogens, DLP4 was recombinantly expressed in *P. pastoris*. Our results showed that DLP4 peptide was expressed as a secretive product with ~7 kDa in GS115. The purified recombinant DLP4 was investigated for its antimicrobial property against Gram-positive bacterium *S. aureus*; Gram-negative bacterium *Salmonella*; and *E. coli*. The antimicrobial assay indicated that the expressed DLP4 was highly active against *S. aureus*, while it exhibited moderate antimicrobial activities against *Salmonella* and *E. coli*.

## 5. Conclusions

This study indicated the potential of BSF larvae in reducing the proliferation of natural zoonotic pathogen populations in pig manure. The results from the qRT-PCR analysis of the AMP genes *DLP4* and *Cg-ubiquitin* and the assessment of the antimicrobial activity of the recombinant DLP4 proved that the BSF larvae possessed AMPs that could functionally inhibit natural zoonotic pathogens. The bacteria from the BSF larval gut, namely, BSF-CL and BSF-F, had broad antagonistic activity against *S. aureus, Salmonella* spp., and *E. coli*. These results suggested that AMPs produced by the BSF larvae and the gut-associated bacteria play a pivotal role in blocking the zoonotic pathogens in pig manure. Our findings suggest that pig manure treated with BSF larvae can be used as an environment-friendly biological process and minimize the environmental impact of pig manure and provide a theoretical basis for further study on the combined mechanism of BSF larvae immunity and its gut microbes against the zoonotic pathogens in pig manure.

## Figures and Tables

**Table 1:** Primer sequences for the quantitative real-time PCR.

Gene	Primer1 Sequence (5′-3′)	Primer 2 Sequence (5′-3′)
qβ-actin	AAACCTTCAACGCCCCAGC	GGCGTGTGGAAGAGCATAACC
qDLP4	CTGTGACCTGTTGAGCCCTTT	AACAGCTCTTTTGTCACACCATC
qCg-Ubiquitin	TCGTCAAGACTTTGACCGGC	GGGTGCGTCCATCTTCCAAT

**Table 2:** Intestinal microorganisms of BSF larvae against *S. aureus*, *E. coli*, and *Salmonella*.

Strain No.	Highest Identity Sequences	Query Coverage	E-Value	Percent Identity	GeneBank Accession No.
BSF-F	*Serratia marcescens*	100%	0.0	100%	CP050960.1
BSF-CL	*B. subtilis*	99%	0.0	99.86%	MW345828.1

**Table 3:** Minimal inhibition concentration (MIC) of DLP4.

Microorganism Strain	MIC (µM)
*Staphylococcus aureus* CICC10001	1.5
*Salmonella enterica* serovar Typhimurium CICC10420	27
*Escherichia coli* CICC10003	>30

## Data Availability

All data are provided in the main body of the published article and Supplementary Material.

## Supplementary Materials

The following supporting information can be downloaded at: https://www.mdpi.com/article/10.3390/insects13010066/s1. Table S1: Primer sequences for the quantitative real-time PCR and recombinant plasmid construction; Strains, plasmids, and reagents; Figure S1: RNA extraction, first-strand cDNA synthesis, and gene amplification; Figure S2: Construction and identification of DLP4 recombinant expression plasmid.
