# Supplementary material for: Inhibition of Zoonotic Pathogens Naturally Found in Pig Manure by Black Soldier Fly Larvae and Their Intestine Bacteria
Source: Insects. 2022 Jan 7;13(1):66. doi: 10.3390/insects13010066 (PMC8779730; doi:10.3390/insects13010066)
Supplement: Supplementary file 1 [file insects-13-00066-s001.zip › insects-1506016-supplementary.pdf]

Supplement information

Table S1. Primer sequences for the quantitative real-time PCR and recombinant plasmid construction

Gene	Primer1 Sequence (5---3)	Primer 2 Sequence (5---3)
qβ-actin	AAACCTTCAACGCCCCAGC	GGCGTGTGGAAGAGC ATAACC
qDLP4	CTGTGACCTGTTGAGCCCTTT	AACAGCTCTTTTGTCA CACCATC
qCg-Ubiquitin	TCGTCAAGACTTTGACCGGC	GGGTGCGTCCATCTT CCAAT
DLP4	CCGCTCGAGAAAAGAGCTAC CTGTGATTTGTTGTC	CGCTCTAGACCCTTTC TGCAGTTGCAGACAGC

1. Strains, plasmids, and reagents

Escherichia coli DH5α, *Escherichia coli* CICC10003 *S. aureus* CICC10001, and *Salmonella enterica* serovar Typhimurium CICC10420 were obtained from the State Key Laboratory of the Agricultural Microbiology of Huazhong Agricultural University in Wuhan, China. *Pichia pastoris* GS115 and the yeast expression vector pGAPZαA were obtained from Invitrogen (USA). The codon-optimized DLP4 gene (120bp) in the pGH vector was provided by TSINGKE Biological Technology (Wuhan, China). TA cloning vector pMD18-T, T4 DNA ligase, dNTPs, DNA Taq polymerase, restriction

endonuclease enzymes, and DNA Marker were purchased from TaKaRa Biotechnology (Japan). AxyPrep plasmid Miniprep Kit and AxyPrep DNA Gel Extraction Kit is the product of Axygen Scientific Inc (USA). Trizol Reagent, RNase free ddH_2O, 4×gDNA wiper, Mix 5× HiScript® II qRT SuperMix, 2×SYBR Green qPCR master mix Kit were the products of Vazyme Biological Technology (Wuhan, China). Diethylpyrocarbonate was obtained from Sigma-Aldrich (St. Louis, USA). Oligonucleotide primers were designed using Primer Premier 5 and synthesized by TSINGKE Biological Technology (Wuhan, China).

2. RNA extraction, first-strand cDNA synthesis, and gene amplification

The total RNA from the BSF larvae was analyzed utilizing a 1% electrophoresis gel (Fig. S1A) and showed three clear bands corresponding to 28S, 18S, and 5S rRNA, without visible degradation. The measured $OD_{260/280}$ values were in the range of 1.8–2, indicating that the purified RNA was of high quality. Moreover, conventional PCR was run for β- actin, DLP4, and Cg-ubiquitin. The amplified gene was analyzed by utilizing 2% electrophoresis gel (Fig. S1B) and sequenced. The PCR results and DNA sequence analysis confirmed that the amplified fragments represented the anticipated cDNA fragment of the tested and control genes.

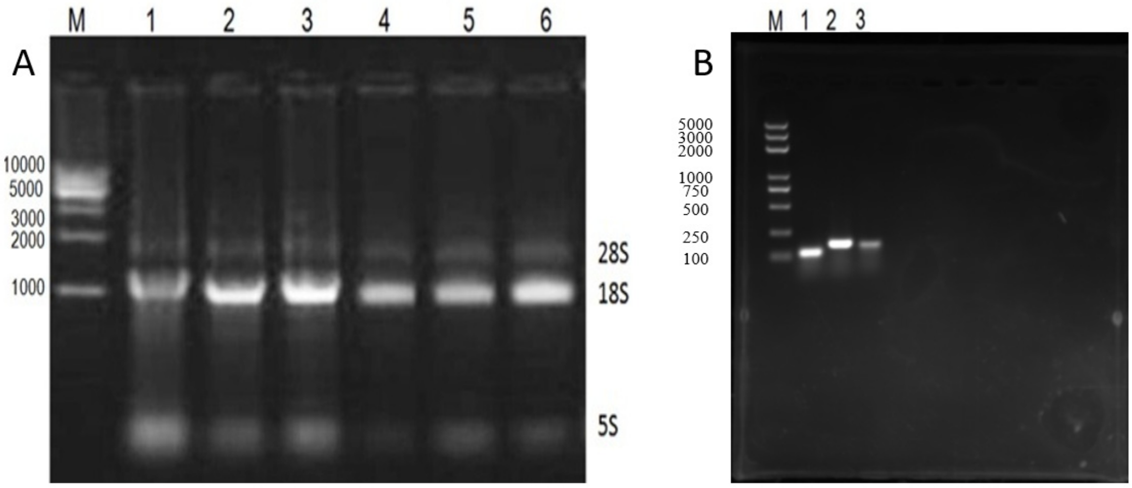

Figure S1. (A) Agarose gel analysis of the extracted RNA from the BSFL. M: 1 kb DNA marker; Lane 1–6 were the extracted total RNA from larvae sampled at 0, 48, 96, 144, 192, and 240 h post-inoculation, respectively. (B) Agarose gel analysis of the PCR products for the tested and control genes from the BSF larvae. M: DL2000 plus DNA marker; Lane 1: DLP4; Lane 2: Cg-ubiquitin; Lane 3 β- actin.

3. Construction and identification of DLP4 recombinant expression plasmid

The DLP4 PCR product with a length of 120 bp (Fig. S2A) was cloned into the PGAPZαA expression vector in-frame to the α-factor secretion signal and downstream of the GAP promoter (Fig. S2B). The constructed plasmid designated as PGAPZαA-DLP4 (Fig. S2C) was analyzed by double digestion by using XhoI and XbaI (Fig. S2D) and then sequenced.

The recombinant plasmid PGAPZαA-DLP4 was linearized by AvrII (Fig. S2E) and then transformed into GS115 competent cells by electroporation. Genomic DNA of the zeocin-resistant transformants was obtained and amplified by PCR by using

pGAP forward and 3AOX1 reverse primers. Electrophoresis analysis of the PCR product revealed the heterologous integration of DLP4 into the host cell genome (Fig. S2F).

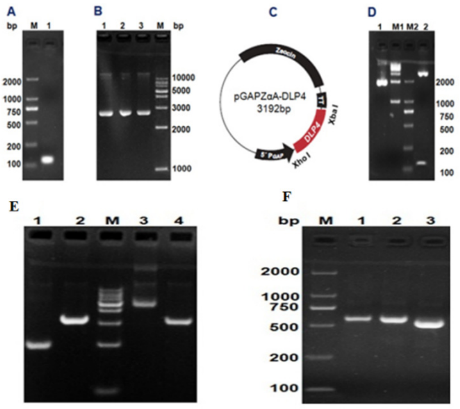

Figure S2. Reconstruction of recombinant plasmid pGAPZαA-DLP4. (A) DLP4 gene amplification. M: 2000 bp DNA marker; Lane 1: the DLP4 ~120 bp. (B) pGAPZαA-DLP4 construction. Lanes 1–3: pGAPZαA-DLP4 recombinant plasmid; Lane M: 1 kb DNA marker. (C) Restriction identification of pGAPZαA-DLP4. (D) Schematic diagram of the *P. pastoris*-expression plasmid, pGAPZαA-DLP4. Lane 1: undigested pGAPZαA; Lane 2: digested pGAPZαA-DLP4 with Xho I and Xba I; M1: 1kb DNA marker; M2: 2000 bp DNA marker. PGAPZαA-DLP4 and pGAPZαA digestion with Avr II and transformant screening. (E) Analysis of pGAPZαA-DLP and pGAPZαA digestion with Avr II. Lane 1: digested pGAPZαA; Lane 2: undigested pGAPZαA. M: 1 kb DNA marker. Lane 3: undigested pGAPZαA-DLP4. Lane 4: digested pGAPZαA-DLP4. (F) DLP4-positive transformant screening by colony PCR. Lane 1: PGAPZαA-

DLP4 positive control; Lanes 2: zeocin-resistant transformants; Lane 3: pGAPZαA containing GS115 cell as a negative control. M: 2000 bp DNA marker.
